# Evaluation of the Impact of Black Carbon on the Worsening of Allergic Respiratory Diseases in the Region of Western Serbia: A Time-Stratified Case-Crossover Study

**DOI:** 10.3390/medicina55060261

**Published:** 2019-06-09

**Authors:** Vesna Tomić-Spirić, Gordana Kovačević, Jelena Marinković, Janko Janković, Anđa Ćirković, Ana Milošević Đerić, Nenad Relić, Slavenka Janković

**Affiliations:** 1 Faculty of Medicine, University of Belgrade, 11000 Belgrade, Serbia; vesnatomicspiric63@gmail.com; 2 Clinic for Allergology and Immunology, Clinical Centre of Serbia, 11000 Belgrade, Serbia; 3 General Hospital Užice, 31000 Užice, Serbia; g.kovacevic.63@gmail.com; 4 Institute of Medical Statistics and Informatics, Faculty of Medicine, University of Belgrade, 11000 Belgrade, Serbia; marinkovic.j@gmail.com; 5 Institute of Social Medicine, Faculty of Medicine, University of Belgrade, 11000 Belgrade, Serbia; drjankojankovic@yahoo.com; 6 Institute of Medical Statistics and Informatics, Faculty of Medicine, University of Belgrade, 11000 Belgrade, Serbia; andja.aleksic@gmail.com; 7 General Hospital Užice, 31000 Užice, Serbia; ana.mdjeric@gmail.com; 8 Department of Otorhinolaryngology, Faculty of Medicine, University of Priština, 38220 Kosovska Mitrovica, Serbia; nenadrelicmd@gmail.com; 9 Institute of Epidemiology, Faculty of Medicine, University of Belgrade, 11000 Belgrade, Serbia

**Keywords:** black carbon, emergency department visits, allergic rhinitis, allergic asthma, case-crossover design, Serbia

## Abstract

*Background and Objectives:* Many epidemiological studies have shown a positive association between black carbon (BC) and the exacerbation of allergic rhinitis and allergic asthma. However, none of the studies in Serbia examined this relationship yet. The aim of this study was to examine the associations between BC and emergency department (ED) visits for allergic rhinitis and allergic asthma in the Užice region of Serbia. *Materials and Methods:* A time-stratified case-crossover design was applied to 523 ED visits for allergic rhinitis and asthma exacerbation that occurred in the Užice region of Serbia between 2012–2014. Data regarding ED visits were routinely collected in the Health Center of Užice. The daily average concentrations of BC were measured by automatic ambient air quality monitoring stations. Odds ratios (ORs) and their corresponding 95% confidence intervals (CIs) were estimated using conditional logistic regression adjusted for the potential confounding influence of weather variables (temperature, humidity, and air pressure). *Results*: Statistically significant associations were observed between ED visits for allergic rhinitis and 2-day lagged exposure to BC (OR = 3.20; CI = 1.00–10.18; *p* = 0.049) and allergic asthma and 3-day lagged exposure to BC (OR = 3.23; CI = 1.05–9.95; *p* = 0.041). *Conclusion:* Exposure to BC in the Užice region increases the risk of ED visits for allergic rhinitis and asthma, particularly during the heating season.

## 1. Introduction

According to the Joint WHO/Convention Task Force on Health Aspects of Air Pollution, black carbon (BC) is a universal indicator of a variable mixture of particulate matter (PM) from a large variety of combustion sources, such as combustion engines, residential burning of wood and coal, and power stations using heavy oil or coal [1]. Atmospheric BC consists of a carbon core enriched with organic compounds from combustion sources [1,2]. BC is known as a better indicator of harmful particulate substances from combustion sources (especially traffic) than undifferentiated PM mass [1] and has a higher association with the incidence of respiratory or cardiovascular diseases per unit (µg/m^3^), as compared to PM with aerodynamic diameters of 2.5 µm or less (PM_2.5_) or of 10 µm or less (PM_10_) [3].

The terms black smoke (BS), elemental carbon (EC), soot, BC, Abs (absorption coefficient) and light-absorbing carbon are used in different studies referring to different methods to measure or express concentrations of BC particles, which is the generic term for any of the different metrics above [1]. 

It is important to distinguish the terms BC and carbon black (CB). Both are formed after the incomplete combustion of hydrocarbons but differ in their constituents and percent carbon contents [4]. While BC is considered as unwanted byproducts derived from incomplete combustion of carbon-containing materials, CB is produced under the controlled conditions in the rubber, printing, and painting industries for commercial use [5,6].

Time-series studies provide sufficient evidence of an association of short-term variations in BC concentrations with short-term changes in all-cause and cardiovascular mortality [7,8], a cardiopulmonary hospital admissions [9,10,11,12]. Cohort studies provide significant evidence of an associations of all-cause, cardiovascular, and respiratory mortality with long-term average BC exposure [13,14,15,16].

Epidemiologic studies indirectly suggest that the inhalation of BC impairs lung function in children. Using the carbon content of airway macrophages as a marker of individual exposure to BC, Kulkarni et al. [17] found direct evidence of this association. The study of Patel et al. [18] indicates that short-term exposure to BC, a diesel exhaust particle indicator, may increase airway inflammation and/or oxidative stress in urban youth and provide mechanistic support for associations documented between BC exposures and respiratory morbidity. In addition, a population-based study showed that occupational exposure to polycyclic aromatic hydrocarbons, a component of soot (i.e., BC), is responsible for respiratory and urinary tract cancers [19]. 

During the latter half of the 20th century, the prevalence of allergic asthma and many other allergic diseases has increased and continues to increase worldwide, particularly in low and middle-income countries [20]. The prevalence of allergic asthma increased from 1996 to 2006 and further to 2016, and it is still ongoing [21]. All studies agree that the prevalence of allergic rhinitis, a condition strongly associated with asthma [22], is still on the rise [23,24].

The International Study of Asthma and Allergy in Childhood (ISAAC) reports demonstrated a wide range of potential risk factors, including outdoor air pollution [25].

The aim of this study was to assess the short-term effect of BC exposure on allergic rhinitis and asthma exacerbations in the Užice region of Western Serbia.

## 2. Materials and Methods

### 2.1. Study Area and Study Population

The study was conducting in the Zlatibor District, Western Serbia, over a two-year period (from 1 July 2012, to 30 June 2014). The main city of the district is Užice, situated on both sides of the river Đetinja, with an average elevation of 411 m above sea level, surrounded by the Dinaric mountains Zlatibor, Tara, and Zlatar. The city of Užice (including Sevojno), with 78,040 inhabitants; Čajetina, with 14,745 inhabitants; and Kosjerić, with 12,090 inhabitants, were included in this study [26]. 

There are three different climates in this region, from moderate-continental to mountain and high-mountain (sub-alpine and alpine) climates. While Užice and Sevojno are heavy industrial centers, the mountain Zlatibor, thanks to the specific continental and Mediterranean air currents, a so-called wind rose, is considered an air spa suitable for the treatment of and recovery from many diseases, including asthma. 

We obtained routinely collected emergency department (ED) visits data for allergic rhinitis, allergic asthma, and allergic asthma with coexisting allergic rhinitis, from the Health Center Užice, either from the EDs (ambulances or home care) in Užice, Sevojno, and Kosjerić, or from a general hospital in Užice. The medical records of all patients were provided retrospectively, and the subjects’ age, gender, diagnosis, and date of visit were recorded. The inclusion criteria were adults aged 18 years and older with the diagnosis of allergic rhinitis (International Classification of Diseases, 10th revision, code J.30.4), allergic asthma (International Classification of Diseases, 10th revision, code J.45.0), or asthma with coexisting allergic rhinitis. Patients with worsening conditions due to respiratory infections or asthma types other than allergic asthma were excluded from this study. 

This study was approved by the Ethics Committee of the Health Center Užice (number of approval: 0303/8785, date of approval: 1 August 2014).

### 2.2. Air Pollutant BC and Weather Variables

Average daily concentrations of BC in micrograms per cubic meter (µg/m^3^) were measured by three automatic ambient air quality monitoring stations located in Užice, Sevojno, and Kosjerić. The BC concentration was measured with reflectometer, a standardized, traditional, and cheap method. According to the OECD standard [27], there is a conversion of the reflectance data into gravimetric units (μg/m^3^).

The daily meteorological dataset (temperature, relative humidity, and surface air pressure) was obtained from the automatic meteorological station located on Zlatibor mountain [28]. 

### 2.3. Study Design and Statistical Analysis 

A time-stratified case-crossover analysis was used to assess the risk of ED visits for allergic rhinitis, asthma, and asthma with coexisting allergic rhinitis due to exposure to BC. The "case-crossover" design is a specific type of case-control study with each case being its own control. This has the advantage as inherent control for potential confounding variables caused by fixed individual characteristics, such as sex and age. "Time-stratified" indicates the method by which the control periods were chosen. In this study, every seventh day before and after the event day was considered a control. The event day is termed lag 0, the day before the event day lag 1, the day before lag 1 is lag 2, and the day before lag 2 is lag 3.

Nominal and ordinal data was described by absolute and relative frequencies, whilst median and interquartile range (IQR) were used for numerical data. To assess differences between groups, Chi-square test and Mann–Whitney test were applied where appropriate.

The degree of association between BC, PM_10_, PM_2.5_ and weather variables was assessed by non-parametric Spearman’s rank correlation.

Conditional logistic regression models were applied for each allergic disease. Different lag periods were included to detect an early (the event day, lag 0), potential delayed (previous three days of exposure, lag 1, 2, and 3, respectively) or cumulative effect of exposure. To control potential confounding factors (i.e., daily weather variables), two models were made: The first model was adjusted for temperature, humidity, and air pressure on the same-day (lag-0); and the second model was adjusted for temperature, temperature^2^, and humidity on the previous day (lag-1). The results of the analyses were expressed as odds ratios (ORs) with their accompanying 95% confidence intervals (CIs). The ORs were calculated in relation to air pollution concentration based on the daily mean level of BC pollutant presented by the IQR. 

A value of *p* < 0.05 was considered statistically significant. All statistical analyses were performed using SPSS version 21.0 software (SPSS Inc., Chicago, IL, USA). 

## 3. Results

A total of 523 ED visits (99 for allergic rhinitis, 179 for allergic asthma alone, and 245 for asthma with allergic rhinitis) occurred during the study period (2012–2014). The mean age of these patients was 45.95 ± 17.24. The majority of patients were female (62.5%) and in the cold seasons (76.3%). More than one third of patients (38%) had one or more comorbidities (such as hypertension, diabetes mellitus, ischemic heart disease, hypothyreosis, hyperthyreosis, or rheumatoid arthritis) (Table 1). 

Daily concentrations of BC and weather variables in the Užice region are shown in Table 2. According to national regulation on monitoring conditions and air quality requirements, daily concentrations of BC exceeded permitted limit values (50 µg/m^3^) during the cold seasons. 

Correlations between BC, PM, and weather variables are shown in Table 3. 

A strong positive correlation was seen between BC and PM_10_ (ρ = 0.75) and between BC and PM_2.5_ (ρ = 0.68), whilst a strong inverse correlation existed between BC and temperature (ρ = 0.67).

**Figure 1:**
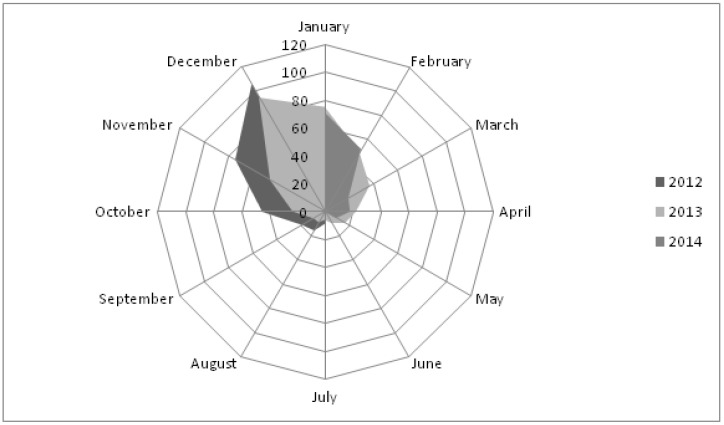
Polar diagram of BC concentration in the air according to months in Užice region, Serbia (from July 2012 to June 2014).

BC concentration was higher during the cold season compared to those during the warm season in all three years of the study period (Figure 1). 

**Figure 2:**
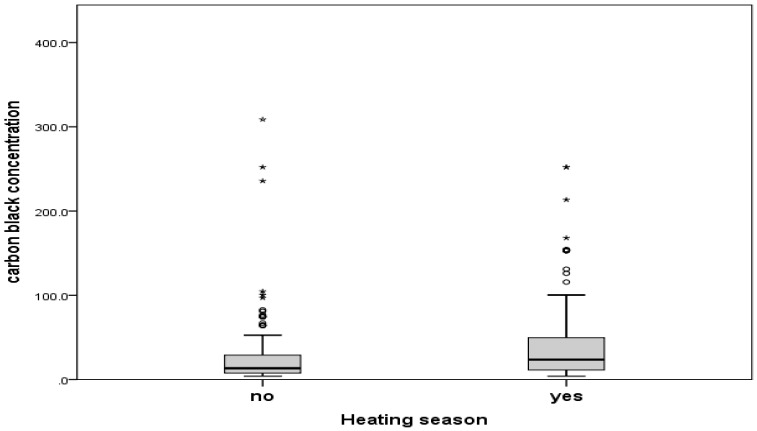
Mean values of BC concentration (µg/m^3^) according to the heating season in Užice region, Serbia (2012–2014).

Moreover, BC concentration was higher during the heating season (Median = 23.67; Min–Max = 4.00–2.52.00) in comparison with the non-heating season (Median = 13.33; Min–Max = 4.00–308.67), and this difference was statistically significant (*p* < 0.001) (Figure 2).

Estimated ORs (crude and adjusted for weather conditions) with 95% CI for ED visits for allergic rhinitis, asthma, and asthma with allergic rhinitis based on a 2-day or 3-day lagged exposure to BC and actual p values are displayed in Table 4.

Statistically significant associations were observed between 2-day lagged exposure to BC and ED visits for allergic rhinitis (ORs = 3.20–3.59; *p* = 0.024–0.049), and between 3-day lagged exposure to BC and ED visits for asthma (ORs = 2.98–3.23; *p* = 0.041–0.048), whilst we failed to find statistically significant associations between BC exposure and asthma with coexisting allergic rhinitis (ORs = 1.57–1.58; *p* < 0.1) (Table 4).

## 4. Discussion

To our knowledge, this is the first study to investigate an association between BC and hospital ED visits for respiratory allergic diseases in Serbia. We performed a time-stratified case-crossover study to assess the effects of the daily concentration of BC on ED visits for allergic rhinitis and allergic asthma after controlling for weather conditions in the Užice region from 2012 to 2014. The results suggest a positive association between ambient exposure to BC and ED visits for allergic rhinitis and asthma. 

We found that IQR concentration of BC in the Užice region increase the risk for allergic rhinitis exacerbation on lag 2 (ORs = 3.20–3.59; CIs = 1.00–10.89; *p* = 0.024–0.049), and asthma exacerbation on lag 3 (ORs = 2.98–3.23; CIs = 1.01–9.95; *p* = 0.041–0.048) more than three-fold. However, we are aware that the actual p values, apart from allergic rhinitis, were very close to 0.05 and considered to be marginal and that caution in interpreting the results is warranted. Our results are in accordance with many previous studies that reported positive associations between BC and ED visits or hospital admissions for asthma [10,11,29,30,31]. Several epidemiologic studies have demonstrated associations between short-term increases in ambient concentrations of EC or BC and increases in respiratory hospital admissions or symptoms [32,33,34,35]. However, Anderson et al. [9] found that respiratory admissions (all ages) were not associated with PM_10_, PM_2.5_, and BS (mainly fine particles of primary origin), except for children in the 0–14 age group, and there were no important seasonal interactions. Recently, Liang et al. [36], using a time-stratified case-crossover method controlled for potential confounders (age, sex, microenvironment, socioeconomic status, nutritional status, and personal habit), found that an IQR increase of BC was associated with a 27.6% (95% CI: 9.6; 48.6) (lag02) increase in respiratory ED visits during a haze season in Beijing. 

Health effects associated with exposure to PM_2.5_ or PM_10_ are usually associated with BC in reviewed epidemiological studies. Effects estimates are much higher for BC compared to PM_2.5_ and PM_10_ when the particulate measures are expressed per unit of mass concentration (μg/m^3^). Janssen et al. [3] and Liang et al. [36] found that on a per μg basis, BC may be more toxic than generic PM_2.5._ These findings are in accordance with the evidence that combustion-related components of PM are more harmful than the non-combustion fractions [37]. As a component of both fine and coarse PM [38], BC may underlie some of the health impacts of PM_2.5._ In our study, positive and strong correlations were observed between BC and both PM_2.5_ and PM_10_ concentrations. Several studies showed that the health effects associated with an IQR increase of BC were similar but not exactly the same with PM_2.5_ [3,36,39,40]. 

It is important to note that current methods of measuring BC and EC need standardization to facilitate comparison between various study results [1].

The results of our study unambiguously indicate that BC was responsible for the acute exacerbation of allergic rhinitis and allergic asthma in the Užice region of Serbia. Considerably greater concentrations of BC in the observed period were recorded in the winter months, as well as during the heating season (from September 15 to April 15) when there were more ED visits due to acute worsening of allergic rhinitis and asthma. A possible explanation for this can be the geographical location of the city of Užice, which is situated in the valley of the river Đetinja above which are the raising hills of the mountain Jelova Gora, whose altitude is 500 m and more above sea level (the bottom of the Užice valley lies at 411 m above sea level near the city beach, and 403 m above sea level at Ada in Krčagovo). 

Above the southern edge of the basin, from the right bank of the river Đetinja, a very steep hill Zabučje rises, with peaks over 700 m above sea level. Therefore, the bottom of the Užice basin is 100 m lower than its surroundings on the north side, and 300 m lower on the southern side. The east-west oriented basin has only one slope that is significantly warmed, and it is the south-oriented side of the mountain Jelova Gora.

Such a terrain configuration significantly influences the creation of local wind systems, especially in combination with common regional winds of low intensity. The south-oriented slopes, which are sunny during the day, emit accumulated heat in the evening, stimulating the circulation of warm air along the slopes and the entry of cooler air into the center of the basin. The winds from the east and west directions ventilate the basin, while winds from the north create stationary vortices, thus preventing basin ventilation. 

During the winter, conditions for temperature inversions are created when cold air falls to the bottom of the basin and a front of warmer air lies above it. With such temperature inversion, vertical air circulation is prevented, so all emitted pollutants are accumulated in the lower layer. Suspended particles, soot, and other air pollutants in winter create a smog that, even when sunny, reflects light, which prevents the warming of the lower air layers and their rise from the basin. At night, the air is further cooled, so the cold air remains trapped in the basin. Consequentially, episodes of high air pollution occur, especially during the heating season in the Užice region. Most days in which the temperature inversion conditions are present are recorded in December and January. Due to global climate change, the number of days with temperature inversion has increased significantly, which is particularly evident in the 2012–2014 years. By analyzing the results, we came to the conclusion that despite the implementation of a series of pollution reduction measures, temperature inversion in high percentage neutralizes the implemented measures.

Furthermore, the Užice architecture, with its characteristic high buildings, also influences air circulation and thus the transport and concentration of the pollutants. Such structures represent obstacles to air flow, as well as surrounding hills above the city. Another effect, so-called a "canyon street effect”, appears in the central streets of the city. This effect makes ventilation difficult or contributes to the creation of vortices in which the air recirculates and holds captured emitted pollutants. Such climatic and topographic characteristics of Užice favor the increased concentration of pollutants emitted, from individual fireplaces, central heating stations, and traffic.

The appearance of thick fogs, resulting in air quality deterioration, is characteristic of the industrial complex in Sevojno and Krčagovo. 

Twenty-eight percent of the total number of households in the central parts of Užice, Krčagovo, and Sevojno, get its heat supply from the city’s central heating plant. This plant, with its 13 boilers rooms in operation, together with individual household boiler rooms and individual fireplaces (which use solid fossil fuels), are one of the largest sources of air pollution in the city. Due to the poor quality of fossil fuels, poor and irregular maintenance of the above-mentioned combustion places, smoke gases emitted into the atmosphere contain harmful and hazardous substances, such as combustion-related particles, known as BC particles. It is estimated that there are around 16,000 individual fireplaces in the city area. Combustion products from these furnaces predominantly remain in the lowest parts of the atmosphere because of relatively low chimneys, the specific configuration of the terrain, and unfavorable air mass flow. The type and quality of energy sources, as well as the combustion process itself, is difficult to modify, because 70% of the inhabitants use wood and coal for heating in individual furnaces. In many household furnaces, completely inappropriate waste materials are used for combustion, like waste oils, textiles, etc., thus further increasing the concentration of pollutants and endangering the environment. Inspection services do not have the legal authority to perform inspection in individual households. 

Considering the geographic position of the Užice, the directions of two main state roads that pass through the central area of the city and a dense network of urban roads and a large volume of traffic through the city center, the traffic in Užice represents a significant source of air pollution, including BC.

### Advantages and Disadvantages of the Study

There are several advantages of our study. Firstly, we studied the novel population. Secondly, the time-stratified case-crossover design used in the present study, in which cases serve as their own control, has been demonstrated as a suitable method for assessing brief changes in risk associated with transient exposures. Also, the reported ORs have been adjusted for the possible confounding influence of weather variables. 

However, there are several methodological limitations. The relatively low number of cases did not allow proper evaluation of potential sex and age differences. Furthermore, the regional measurements of BC pollution from fixed-site monitoring stations were taken as the measurements of exposure to BC pollutant for each individual in this study. In addition, we did not adjust for the confounding influence of the levels of other air pollutants and aeroallergens that could lead to a change in risk. 

## 5. Conclusions

Taking into consideration all limitations, our study supports the association between exposure to BC and ED visits for allergic rhinitis and allergic asthma in the Užice region of Western Serbia. Considering the importance of the geographical location of the study area as a combination of an industrial region and a climatic health resort suitable for the treatment of respiratory diseases, the results of our study are of great public health importance. The analysis of the short-term effect of outdoor BC on allergic rhinitis and allergic asthma exacerbation in the Užice region may significantly contribute to the establishment of relevant public policy in Western Serbia. 

According to WHO recommendations [41], particulate air pollution, including BC, can be reduced by using stricter air quality standards and limits for emissions from various sources, by reducing energy consumption (especially those based on combustion sources), and by changing modes of transport (i.e., reducing the number of motor vehicles that pass through the center of the city, using the surrounding roads and one-way streets, etc.) and individual behavior (i.e., using cleaner modes of transport, excluding coal as a means of heating in households, etc.). Reasonable efforts to reduce the level of BC concentration could decrease the occurrence of allergic rhinitis and asthma, as well as their exacerbation in the Užice region.

In addition, we hope that the present study provides evidence to support the regular monitoring of BC and PM_2.5_ in the Užice region. Because of the environmental health risks associated with BC, understanding exposure levels to BC and regulations to reduce total BC emissions is a high priority of the Serbian Environmental Protection Agency and other stakeholders in Serbia.

## Figures and Tables

**Table 1:** Characteristics of asthma emergency department (ED) visits (523) in Užice region, Serbia (2012–2014).

Variables	Allergic Rhinitis	Asthma	Asthma with AR	Total
	*n* = 99	*n* = 179	*n* = 245	*n* = 523
**Age** (years ± SD)	41.43 ± 18.00	50.15 ± 17.60	44.71 ± 16.03	45.95 (17.24)
**Sex**				
Male (*n*, %)	57 (57.6)	52 (29.1)	87 (35.5)	196 (37.5)
Female (*n*, %)	42 (42.4)	127 (70.9)	158 (64.5)	327 (62.5)
**Season**				
Worm (*n*, %)	25 (25.3)	36 (20.1)	58 (23.7)	119 (22.8)
Cold (*n*, %)	74 (74.7)	143 (79.9)	187 (76.3)	404 (76.3)
**Comorbidities**				
0 (*n*, %)	78 (78.8)	101 (56.4)	144 (58.8)	323 (61.8)
1 (*n*, %)	10 (10.1)	52 (29.1)	71 (29.1)	133 (25.5)
≥2 (*n*, %)	11 (11.1)	26 (14.5)	29 (11.9)	66 (12.6)

AR: allergic rhinitis.

**Table 2:** Daily concentrations of black carbon and weather variables according to seasons in Užice region, Serbia (2012–2014).

Variable	Mean	SD	Min	Max	Median	IQR	
						P25	P75
**BC (µg/m^3^)**							
Total	33.87	35.59	3.33	308.66	36.00	10.00	46.00
Spring	15.81	8.63	4.00	45.00	13.25	8.33	21.58
Summer	10.55	5.82	3.33	30.00	6.33	6.00	12.33
Autumn	47.94	35.74	7.33	240.00	34.08	25.58	59.67
Winter	63.02	42.90	10.00	308.66	41.58	35.67	77.25
**Temperature (°C)**							
Total	15.80	9.84	−6.66	36.23	16.46	7.26	23.73
Spring	17.89	6.52	1.33	32.60	8.12	14.26	22.38
Summer	26.17	5.26	10.46	36.23	7.37	22.93	30.30
Autumn	12.30	8.65	−6.66	32.43	19.32	5.14	19.45
Winter	6.25	5.29	−3.40	20.07	7.42	2.50	9.91
**Humidity (%)**							
Total	56.42	20.51	11.53	98.80	34.46	38.80	73.26
Spring	52.48	18.42	16.67	93.53	32.35	37.00	69.35
Summer	40.34	15.19	11.53	93.20	17.73	29.73	47.46
Autumn	64.27	17.84	22.43	98.50	17.84	50.69	79.66
Winter	69.52	17.08	24.00	98.80	23.48	60.16	83.63
**Air pressure (mbar)**							
Total	962.70	6.68	936.33	984.00	7.86	958.93	966.80
Spring	960.68	5.36	946.00	974.00	7.28	956.88	964.14
Summer	963.64	3.36	947.23	971.50	4.63	961.50	966.13
Autumn	964.76	7.78	942.33	984.00	10.07	960.04	970.11
Winter	961.74	8.28	936.33	982.90	11.47	956.26	967.73

IQR: Interquartile range; BC: Black carbon.

**Table 3:** Correlation coefficients with *p* values* between PM_2.5_, PM_10_, BC, and weather variables in Užice region, Serbia (2012–2014).

Variable	PM_2.5_	PM_10_	BC	Temperature	Humidity
PM_2.5_	1.00				
PM_10_	**0.83**	**1.00**			
	*p* < 0.001				
BC	**0.68**	**0.75**	**1.00**		
	*p* < 0.001				
Temperature	**−0.56**	**−0.54**	**−0.67**	**1.00**	
	*p* < 0.001	*p* < 0.001	*p* < 0.001		
Humidity	**0.33**	**0.28**	**0.41**	**−0.77**	**1.00**
	*p* < 0.001	*p* < 0.001	*p* < 0.001	*p* < 0.001	
Air pressure	0.08	0.15	0.01	0.06	−0.14
	*p* = 0.002	*p* < 0.001	*p* = 0.736	*p* = 0.011	*p* < 0.001

* Spearman correlation coefficients. PM_2.5_: particulate matter with an aerodynamic diameter of 2.5 µm or less; PM_10_: particulate matter with an aerodynamic diameter of 10 µm or less; BC: Black carbon. Bold values are statistically significant.

**Table 4:** Relationship between exposure to BC and ED visits for allergic rhinitis and asthma in Užice region, Serbia (2012–2014).

ED visits	Lags	OR * (95% CI), *p*	OR ^†^ (95% CI), *p*	OR ^‡^ (95% CI), *p*
Allergic rhinitis	2-day lag	**3.59 (1.18–10.89),** **0.024**	**3.20 (1.00–10.18),** **0.049**	**3.24 (1.03–10.22),** **0.045**
Asthma	3-day lag	**2.98 (1.01–8.82),** **0.048**	**3.23 (1.05–9.95),** **0.041**	**2.98 (1.01–8.82),** **0.048**
Asthma with allergic rhinitis	3-day lag	1.58 (0.95–2.64), 0.078	1.57 (0.97–2.54), 0.067	1.57 (0.97–2.54), 0.067

ED: Emergency department. OR: Odds ratio; CI: Confidence intervals. * Unadjusted. ^†^ Adjusted for temperature, humidity, and air pressure on the same day. ^‡^ Adjusted for temperature, temperature^2^, and humidity on the previous day. Odds ratios were calculated for the IQR of BC concentration. Statistically significant values are in bold.

## References

[1] WHO Health Effects of Black Carbon. Policy Implications for Countries in Eastern Europe, Caucasus and Central Asia. Copenhagen: World Health Organization Regional Office for Europe, 2012. http://www.euro.who.int/__data/assets/pdf_file/0004/162535/e96541.pdf.

[2] Air Quality Expert Group (2005). What is causing the health effects of particles?. Particulate Matter in the UK: Summary.

[3] Janssen N.A., Hoek G., Simic-Lawson M., Fischer P., van Bree L., ten Brink H., Keuken M., Atkinson R.W., Anderson H.R., Brunekreef B. (2011). Black carbon as an additional indicator of the adverse health effects of airborne particles compared with PM_10_ and PM_2.5_. Environ. Health Perspect..

[4] Niranjan R., Thakur A.K. (2017). The Toxicological Mechanisms of Environmental Soot (Black Carbon) and Carbon Black: Focus on Oxidative Stress and Inflammatory Pathways. Front. Immunol..

[5] Long C.M., Nascarella M.A., Valberg P.A. (2013). Carbon black vs. black carbon and other airborne materials containing elemental carbon: Physical and chemical distinctions. Environ. Pollut..

[6] Watson A.Y., Valberg P.A. (2001). Carbon black and soot: Two different substances. AIHAJ.

[7] Bremner S.A., Anderson H.R., Atkinson R.W., McMichael A.J., Strachan D.P., Bland J.M., Bower J.S. (1999). Short-term associations between outdoor air pollution and mortality in London 1992–1994. Occup. Environ. Med..

[8] Hoek G., Brunekreef B., Verhoeff A., van Wijnen J., Fischer P. (2000). Daily mortality and air pollution in The Netherlands. J. Air Waste Manag. Assoc..

[9] Anderson H.R., Bremner S.A., Atkinson R.W., Harrison R.M., Walters S. (2001). Particulate matter and daily mortality and hospital admissions in the West Midlands conurbation of the United Kingdom: Associations with fine and coarse particles, black smoke and sulphate. Occup. Environ. Med..

[10] Atkinson R.W., Anderson H.R., Strachan D.P., Bland J.M., Bremner S.A., Ponce de Leon A. (1999). Short-term associations between outdoor air pollution and visits to accident and emergency departments in London for respiratory complaints. Eur. Respir. J..

[11] Atkinson R.W., Bremner S.A., Anderson H.R., Strachan D.P., Bland J.M., de Leon A.P. (1999). Short-term associations between emergency hospital admissions for respiratory and cardiovascular disease and outdoor air pollution in London. Arch. Environ. Health.

[12] Le Tertre A., Medina S., Samoli E., Forsberg B., Michelozzi P., Boumghar A., Vonk J.M., Bellini A., Atkinson R., Ayres J.G. (2002). Short-term effects of particulate air pollution on cardiovascular diseases in eight European cities. J. Epidemiol. Community Health.

[13] Smith K.R., Jerrett M., Anderson H.R., Burnett R.T., Stone V., Derwent R., Atkinson R.W., Cohen A., Shonkoff S.B., Krewski D. (2009). Public health benefits of strategies to reduce greenhouse-gas emissions: Health implications of short-lived greenhouse pollutants. Lancet.

[14] Lipfert F.W., Baty J.D., Miller J.P., Wyzga R.E. (2006). PM_2.5_ constituents and related air quality variables as predictors of survival in a cohort of US military veterans. Inhal. Toxicol..

[15] Beelen R., Hoek G., van den Brandt P.A., Goldbohm R.A., Fischer P., Schouten L.J., Jerrett M., Hughes E., Armstrong B., Brunekreef B. (2008). Long-term effects of traffic-related air pollution on mortality in a Dutch cohort (NLCS-AIR study). Environ. Health Perspect..

[16] Filleul L., Rondeau V., Vandentorren S., Le Moual N., Cantagrel A., Annesi-Maesano I., Charpin D., Declercq C., Neukirch F., Paris C. (2005). Twenty five year mortality and air pollution: Results from the French PAARC survey. Occup. Environ. Med..

[17] Kulkarni N., Pierse N., Rushton L., Grigg J. (2006). Carbon in Airway Macrophages and Lung Function in Children. N. Engl. J. Med..

[18] Patel M.M., Chillrud S.N., Deepti K., Ross J.M., Kinney P.L. (2013). Traffic-related air pollutants and exhaled markers of airway inflammation and oxidative stress in New York City adolescents. Environ. Res..

[19] Bosetti C., Boffetta P., La Vecchia C. (2007). Occupational exposures to polycyclic aromatic hydrocarbons, and respiratory and urinary tract cancers: A quantitative review to 2005. Ann. Oncol..

[20] Pawankar R. (2014). Allergic diseases and asthma: A global public health concern and a call to action. World Allergy Organ. J..

[21] Backman H., Räisänen P., Hedman L., Stridsman C., Andersson M., Lindberg A., Lundbäck B., Rönmark E. (2017). Increased prevalence of allergic asthma from 1996 to 2006 and further to 2016—Results from three population surveys. Clin. Exp. Allergy.

[22] Braunstahl G.J. (2009). United airways concept: What does it teach us about systemic inflammation in airways disease?. Proc. Am. Thorac. Soc..

[23] Asher M.I., Montefort S., Björkstén B., Lai C.K., Strachan D.P., Weiland S.K., Williams H., ISAAC Phase Three Study Group (2006). Worldwide time trends in the prevalence of symptoms of asthma, allergic rhinoconjunctivitis, and eczema in childhood: ISAAC Phases One and Three repeat multicountry cross-sectional surveys. Lancet.

[24] Simpson C.R., Newton J., Hippisley-Cox J., Sheikh A. (2008). Incidence and prevalence of multiple allergic disorders recorded in a national primary care database. J. R. Soc. Med..

[25] Brunekreef B., Stewart A.W., Anderson H.R., Lai C.K., Strachan D.P., Pearce N., ISAAC Phase 3 Study Group (2009). Self-reported truck traffic on the street of residence and symptoms of asthma and allergic disease: A global relationship in ISAAC phase 3. Environ. Health Perspect..

[26] (2014). Census of Population, Households and Dwellings in the Republic of Serbia 2011: Comparative Overview of the Number of Population in 1948, 1953, 1961, 1971, 1981, 1991, 2002 and 2011, Data by Settlements.

[27] OECD (1964). Methods of Measuring Air Pollution. Report of the Working Party on Methods of Measuring Air Pollution and Survey Techniques.

[28] National Network of Automatic Stations for Air Quality Monitoring. http://www.amskv.sepa.gov.rs/?lng=en.

[29] Walters S., Griffiths R.K., Ayres J.G. (1994). Temporal association between hospital admissions for asthma in Birmingham and ambient levels of sulphur dioxide and smoke. Thorax.

[30] Castellsague J., Sunyer J., Sáez M., Antó J.M. (1995). Short-term association between air pollution and emergency room visits for asthma in Barcelona. Thorax.

[31] Sunyer J., Spix C., Quénel P., Ponce-de-León A., Pönka A., Barumandzadeh T., Touloumi G., Bacharova L., Wojtyniak B., Vonk J. (1997). Urban air pollution and emergency admissions for asthma in four European cities: The APHEA Project. Thorax.

[32] Bell M.L., Ebisu K., Peng R.D., Samet J.M., Dominici F. (2009). Hospital admissions and chemical composition of fine particle air pollution. Am. J. Respir. Crit. Care Med..

[33] Gent J.F., Koutrakis P., Belanger K., Triche E., Holford T.R., Bracken M.B., Leaderer B.P. (2009). Symptoms and medication use in children with asthma and traffic-related sources of fine particle pollution. Environ. Health. Perspect..

[34] Patel M.M., Chillrud S.N., Correa J.C., Hazi Y., Feinberg M., Kc D., Prakash S., Ross J.M., Levy D., Kinney P.L. (2010). Traffic-related particulate matter and acute respiratory symptoms among New York City area adolescents. Environ. Health Perspect..

[35] Spira-Cohen A., Chen L.C., Kendall M., Lall R., Thurston G.D. (2011). Personal exposures to traffic-related air pollution and acute respiratory health among Bronx school children with asthma. Environ. Health Perspect..

[36] Liang F., Tian L., Guo Q., Westerdahl D., Liu Y., Jin X., Li G., Pan X. (2017). Associations of PM_2.5_ and Black Carbon with Hospital Emergency Room Visits during Heavy Haze Events: A Case Study in Beijing, China. Int. J. Environ. Res. Public Health.

[37] Krzyzanowski M., Kuna-Dibbert B., Schneider J. (2005). Health Effects of Transport-Related Air Pollution.

[38] Viidanoja J., Sillanpää M., Laakia J., Kerminen V.M., Hillamo R., Aarnio P., Koskentalo T. (2002). Organic and black carbon in PM_2.5_ and PM_10_: 1 year of data from an urban site in Helsinki, Finland. Atmos. Environ..

[39] Zanobetti A., Schwartz J. (2006). Air pollution and emergency admissions in Boston, MA. J. Epidemiol. Community Health.

[40] Wang X., Chen R.J., Meng X., Geng F.H., Wang C.C., Kan H.D. (2013). Associations between fine particle, coarse particle, black carbon and hospital visits in a Chinese city. Sci. Total Environ..

[41] WHO Health Effects of Particulate Matter. Policy Implications for Countries in Eastern Europe, Caucasus and Central Asia. Copenhagen: World Health Organization Regional Office for Europe, 2013. http://www.euro.who.int/__data/assets/pdf_file/0006/189051/Health-effects-of-particulate-matter-final-Eng.pdf.

